# Exploring the Aortic Root Diameter and Left Ventricle Size among Lithuanian Athletes

**DOI:** 10.3390/medicina55060271

**Published:** 2019-06-11

**Authors:** Renata Žumbakytė-Šermukšnienė, Agnė Slapšinskaitė, Miglė Baranauskaitė, Julija Borkytė, Rasa Sederevičiūtė, Kristina Berškienė

**Affiliations:** 1 Sports Medicine Clinics, Lithuanian University of Health Sciences, LT-47181 Kaunas, Lithuania; agne.slapsinskaite@gmail.com (A.S.); migkrep@gmail.com (M.B.); borkytejulija@gmail.com (J.B.); k.berskiene@gmail.com (K.B.); 2 Health Research Institute, Lithuanian University of Health Sciences, LT-47181 Kaunas, Lithuania; 3 Radiology Clinic, Lithuanian University of Health Sciences, LT-50161 Kaunas, Lithuania; sedererasa@yahoo.com

**Keywords:** performance, sports, aortic valve, sinus of Valsalva, echocardiography, sudden cardiac death

## Abstract

*Background and objectives*: Aortic rupture is known as one of the potential causes of sudden cardiac death in athletes. Nevertheless, adaptation strategies for aortic root dilation in athletes vary. The purpose of this study was to investigate aortic root adaptation to physical workload and to determine if aortic roots and left ventricle sizes are contingent upon the physical workload. *Materials and Methods*: Echocardiography was applied to 151 subjects to measure the aortic root at aortic valve annulus (AA) and at sinus of Valsalva (VS). 122 were athletes (41 females and 81 males) and 29 were non-athletes (14 females and 15 males). Of the 41 female athletes, 32 were endurance athletes, and 9 were strength athletes. From 81 male athletes, 56 were endurance athletes, and 25 were strength athletes. AA and VS mean values for the body surface area were presented as AA relative index with body surface area (rAA) and VS relative index with body surface area (rVS). Left ventricle (LV) measures included LV end-diastolic diameter (LVEDD), interventricular septum thickness in diastole (IVSTd), LV posterior wall thickness in diastole (LVPWTd), LV mass (LVM), LV mass index, and LV end-diastolic diameter index (LVEDDI). *Results*: Results indicated that VS was higher in female athletes (28.9 ± 2.36 mm) than in non-athletes (27.19 ± 2.87 mm, *p* = 0.03). On the other hand, rAA was higher in strength athletes (12.19 ± 1.48 mm/m^2^) than in endurance athletes (11.12 ± 0.99 mm/m^2^, *p* = 0.04). Additionally, rVS and rAA were higher in female strength athletes (17.19 ± 1.78 mm/m^2^, 12.19 ± 1.48 mm/m^2^*)* than female basketball players (15.49 ± 1.08 mm/m^2^, *p* = 0.03, 10.75 ± 1.06 mm/m^2^, *p* = 0.02). No significant differences regarding aortic root were found between male athletes and non-athletes. Statistically significant positive moderate correlations were found between VS and LVEDD, LVM, IVSTd, LVPWTd, rVS, and LVEDDI parameters in all athletes. *Conclusion*: The diameter of Valsalva sinus was greater in female athletes compared to non-athletes. The rAA mean value for body surface area was greater in female athletes practising strength sports as compared to their counterparts who were practising endurance sports. The diameter of the aortic root at sinuses positively correlated with the LV size in all athletes.

## 1. Introduction

Regular physical activity has many beneficial effects on the reduction of ischemic heart disease and myocardial infarction, however participation in competitive sports always involves a likelihood of sudden death. The specific focus on healthy young athletes is highly understandable given the substantial social and emotional impact of sudden and unexpected deaths in this group. In novices and professional athletes sudden cardiac death is not common, however, extremely visible [1]. In sudden death of athletes, the role of aortic root should not be forgotten given the fact that aortic stenosis is well-known as an important cause of young athletes’ death. Importantly, aortic dissection/rupture is a catastrophic condition not usually associated with demise early in life [2].

Recent developments in football have seen the sudden death of young football players due to aortic rupture, hence reinforcing the controversy of football as a field with substantial risk for sudden cardiac arrest and death [3,4,5,6,7]. Moreover, there is an argument that aortic dilatation and the subsequent event of thoracic aortic aneurysm may be an occupational disease due to the nature of some vocations (i.e., military and security personnel, blue collar workers, weightlifters, athletes, etc.) [8]. Of particular importance, there is some evidence that elite athletic training is associated with small, but significantly larger, aortic root diameter (i.e., sinuses of Valsalva) [9]. Surprisingly, adaptation strategies for aortic root dilation in athletes still vary to a large extent [10].

To that end, the differential impact of distinct forms of exercise on arteries in strength athletes versus endurance athletes have not been investigated [11]. Further advancement of this topic could be of particular interest for clinicians. For instance, a consensus or informed recommendation for clinicians facing cases of higher aortic indices, with higher body surface area and with or without remodelled left ventricle (LV), were called upon [11].

Of interest to this study, it is known that endurance sports can lead to volume overload and an increase in the diameter of the heart chambers. Leischik and Spelsberg [12] demonstrated a relationship between myocardial thickening and exercise arterial pressure during endurance exercise. Importantly, raised blood pressure can lead to pathological enlargement of atrial dimensions in athletes [13]. The enlargement of the left atrium may further lead to atrial fibrillation and higher activity of electric circuits [13]. Strength sports can cause pressure overload and are particularly related to the thickening of the LV wall [14]. While “athlete’s heart” is a long investigated topic within sports medicine, Green et al. have provided some insights into the functioning of “athlete’s artery” [11]. In fact, more information into cardiac pathophysiological training-caused adaptation is needed. Increased understanding of this adaptation could indeed help reduce risk for sudden cardiac arrest and death and/or irreversible cardiac damage identification and this importance [15].

Zeppill et al. [16] were among the first ones to report a wider diameter of arteries and veins in endurance athletes compared to non-athletes. Similarly, others have also suggested significant differences between arteries in athletes and non-athletes [11]. Nevertheless, findings from work investigating aortic root vessels were controversial and inconsistent. Specifically, Pelliccia et al. [17] found significant differences between endurance athletes and the control group. However, while endurance athletes had higher diameters in aorta compared to non-athletes, none of these results were replicated in strength athletes.

In fact, LV remodelling work has indicated LV association with physical activity type. However, to date, little is known about if the changes of the aortic root are linked with LV size. Specifically, clinicians face an issue of whether to set higher aortic indices for athletes with a higher body surface area and with or without remodelled LV (i.e., basketball players, strength athletes) in cases when relative values are normal. Thus, interpretation of increased rates of aortic indices with normal relative values is challenging, especially among non-athletes, overweight, and obese individuals. To further complicate things, it is also known that for physically inactive persons with metabolic syndrome, LV remodelling and blood pressure are co-occurring with hypertension.

In the present study, we evaluated the development of the aortic root diameter with blood pressure, LV size, and physical activity type. Consequently, the purpose of this study was to investigate aortic root adaptation to physical workload and to determine whether the aortic root’s size and left ventricle remodelling is dependent on physical load. Drawing upon previous studies, we hypothesized that the cardiovascular system’s adaptation to physical load will be presented through changes in aortic root’s size, dependent on LV remodelling. In other words, we expected that when athletes’ LV would not be remodelled, no changes would occur in the aortic root’s size either.

## 2. Materials and Methods

### 2.1. Sample

Preliminary data for retrospective study was collected from a total of 944 subjects in Kaunas Department of Lithuanian Sports Medicine Centre during the recruiting period in 2014–2015. All the experimental procedures were approved by Kaunas Regional Biomedical Research Ethics Committee (No.BE-2-14) on January 21 2019 and conducted in accordance with the Declaration of Helsinki. Caucasian subjects who met the inclusion criteria. The inclusion criteria were as follows:

- A total of two-dimensional (2D) transthoracic echocardiography performed in Kaunas Department of Lithuanian Sports Medicine Centre 2014-2015.
- Age range 16–35 years, given the literature definition of “young” and “old” athletes as <35 and >35 years [3].
- Physical activity levels (athletes or non-athletes). Individuals who participated in sports for more than 4 years and 4.5 h per week were included in the group of athletes. Individuals who were active for less than 4 years and/or 4.5 h per week were classified as non-athletes [18].
- Physical activity type (i.e., endurance and strength sports).
- No current diagnosis or previous history about cardiovascular diseases (for instance, bicuspid aortic valves, Marfan syndrome, patent ductus arteriosus, aortic coarctation).
- No activity on the test day.

Individuals who did not meet one or more of the inclusion criteria were excluded from the study. The subjects were divided into groups according to gender (i.e., female, male), physical activity (i.e., athletes, non-athletes), and physical activity type (i.e., strength and endurance sports). Of the 151 subjects, 122 were athletes (41 females and 81 males) and 29 were non-athletes (14 females and 15 males). Of the 41 female athletes, 32 were endurance athletes, and 9 were strength athletes. From 81 male athletes, 56 were endurance athletes, and 25 were strength athletes. To that end, 12 female and 19 male basketball players took part in the study (see Table 1, Table 2, and Table 3). Based on Pelliccia et al. [19] proposed sports disciplines classification, we grouped 122 athletes to endurance and power groups. According to this classification, basketball is placed in the mixed group. Further, Pelliccia et al. [19] states that power, endurance, and mixed sport disciplines mostly influences the cardiac remodeling. Of importance herein, we expected that a large body surface area of the basketball players may have an effect to the relative values of the aortic root diameter. According to Kinoshita et al. [20], a nonlinear relationship with plateau between aortic root dimensions and body surface area (BSA) was observed in a study of 1929 Japanese athletes, of whom 415 (>20%) participated in either basketball or volleyball and had anthropometric measures significantly greater than those of the other sports athletes [9]. This nonlinear relationship between anthropometric and aortic measurements, which suggests a plateauing of aortic dimensions with increasing height and body surface area, further emphasizes the importance of not dismissing markedly enlarged aortas in large athletes as a result of athletic training or body size alone [9]. Due to the aforementioned reasoning, the basketball players were separated from other endurance and power athletes.

### 2.2. Protocol

All subjects underwent two-dimensional (2D) transthoracic echocardiography (TTE) procedure. Prior to performing 2D TTE, subjects’ arterial blood pressure [21], heart rate, height, weight, and self-reported physical activity levels were measured.

The ultrasound system CX50 (Philips Ultrasound, Bothell, WA, USA), with transducer S5-1, was used in this study. Two physicians performed 2D TTE and averages for all variables of interest were computed. The measurements of aortic root and the left ventricle were drawn upon the guidelines of the American society of echocardiography and the European association of cardiovascular imaging [22]. The maximal diameter of the sinuses of Valsalva was measured at end-diastole, in a strictly perpendicular plane to that of the long axis of the aorta using the edge to leading edge (L-L) convention. The aortic annulus was measured at mid-systole from inner edge to inner edge (I-I). This was done in order to obtain the rounder shape and bigger diameter of aortic annulus. All measurements were taken in the perspective that depicts the maximum aortic diameter perpendicular to the long axis of the aorta. Taking into account that aortic root diameter measurements at the level of the sinuses of Valsalva are closely related to body surface area (BSA) and age [22], aortic dilatation can be easily detected by plotting observed aortic root diameter versus BSA on previously published nomograms [22,23].

Due to the significant difference (*p* < 0.05) between the subjects’ height and weight in the groups of athletes and non-athletes, we evaluated the relative indices with the area of the body surfaces. Relative body surface area was calculated by means of Mosteller’s (1987) formula [24].

The following dimensions of the root of the aorta were evaluated as follows: sinuses of Valsalva (VS, mm) and its relative index with body surface area (rVS, mm/m^2^), aortic valve annulus (AA, mm) and its relative index with body surface area (rAA, mm/m^2^).

For left ventricle (LV) measurements, the following were used: LV end-diastolic diameter (LVEDD, mm), interventricular septum thickness in diastole (IVSTd, mm), and LV posterior wall thickness in diastole (LVPWTd, mm). Subsequently, we computed: LV mass (LVM, g), LV mass index (LVMI, g/m^2^), and LV end-diastolic diameter index (LVEDDI, mm/m^2^). The formula for computing the mass of the LV was drawn upon the most recent recommendations of the American society of echocardiography and the European cardiovascular association [18].

To determine the potential association between aorta root size and the size of the LV and blood pressure, we computed correlational analyses between VS and LV size: LVEDD, LVM, IVSTd, and LVPWTd. As well as systolic blood pressure and its correlation with rVS, LVEDDI, and LVMI. The quantitative values are given as the arithmetic mean ± standard deviation (x ± SD). Student t test was used to compare the independent samples. The correlation coefficient (*r*) of Spearman was used for assessing the direction and strength between variables. A |*r*| < 0.3 was considered weak, while 0.3 ≤ |*r*| ≤ 0.7 was considered as moderate and |*r*| > 0.7 was considered strong. We deemed a *p* value less than 0.05 to be significant.

## 3. Results

### 3.1. Aortic Root Diameter

In our study, VS was higher in female athletes (28.9 ± 2.36 mm) compared to non-athletes (27.19 ± 2.87 mm, *p* = 0.03). When comparing rVS, there was no significant difference in female athletes (16.19 ± 1.59 mm/m^2^) compared to non-athletes (15.84 ± 1.98 mm/m^2^, *p* = 0.44). There were no significant differences between the female endurance athletes (28.99 ± 2.32 mm) and strength athletes 28.56 ± 2.64 mm (*p* = 0.48) with regards to VS. Moreover, while comparing relative measurements with body surface area, there were no significant differences between female endurance athletes (15.90 ± 1.44 mm/m^2^) and female strength athletes (17.19 ± 1.78 mm/m^2^, *p* = 0.06) with regards to rVS.

There were no significant differences of AA diameter between female endurance athletes and strength athletes. However, rAA was significantly higher in female strength athletes than female endurance athletes (12.19 ± 1.48 mm/m^2^ versus 11.12 ± 0.99 mm/m^2^, *p* = 0.04).

There were significant differences between the relative values of the aortic root, but not at the AA nor VS between basketball players and the strength athletes. However, statistically significant differences were observed (*p* = 0.03) in rVS between strength athletes (17.19 ± 1.78 mm/m^2^) and female basketball players (15.49 ± 1.08 mm/m^2^). rAA in female strength athletes (12.19 ± 1.48 mm/m^2^) was higher than in the female basketball players (10.75 ± 1.06 mm/m^2^, *p* = 0.02).

There were no significant differences with regards to the aortic root at VS between male athletes and non-athletes (VS was 32.38 ± 2.96 mm, rVS was 16.11 ± 1.67 mm/m^2^ and VS was 31.39 ± 2.72 mm, *p* = 0.14, rVS was 15.80 ± 1.58 mm/m^2^, *p* = 0.35, respectively). Similarly, no significant differences were obtained with regards to aortic root at VS in male endurance athletes and strength athletes (VS was 32.08 ± 3.01 mm, rVS was 16.00 ± 1.63 mm/m^2^ and VS was 33.02 ± 2.80 mm, *p* = 0.20, rVS was 16.34 ± 1.75 mm/m^2^, *p* = 0.32, respectively). Finally, with regards to aortic root at VS in male athletes, there were no significant differences between basketball players and strength athletes (VS was 15.47 ± 1.38 mm/m^2^ and VS was 16.34 ± 1.75 mm/m^2^, *p* = 0.11, respectively).

### 3.2. Aortic Root Diameter Correlates with Left Ventricle Size

A significant positive moderate correlation was found between VS and LVM, as well as rVS and LVEDDI in female and male athletes (see Table 4). Additionally, a significant moderate correlation (*r* = 0.32, *p* = 0.04) was found between VS and LVEDD in female athletes. Moreover, in female athletes, a statistically significant positive moderate correlation (*r* = 0.54, *p* <0.001) was found between the VS and IVSTd. Similarly, a statistically significant positive moderate correlation (*r* = 0.57, *p* <0.001) was found between VS and LVPWTd in female athletes. Statistically significant strong correlations between VS and LVM (*r* = 0.76, *p* = 0.02), rVS, and LVEDDI (*r* = 0.73, *p* = 0.03) parameters were also found in female strength athletes. Finally, a statistically significant moderate correlation (*r* = 0.69, *p* = 0.04) was found between VS and LVPWTd.

Significant and moderate correlations (*r* = 0.32, *p* = 0.004), (*r* = 0.37, *p* = 0.001), and (*r* = 0.37, *p* = 0.001), were found between VS and LVEDD, VS and IVSTd, and VS and LVPWTd, respectively, in male athletes. In male endurance athletes, moderate correlations between VS and LVEDD (*r* = 0.38, *p* = 0.004), as well as between VS and IVSTd (*r* = 0.39, *p* = 0.003), were shown. Furthermore, the statistically significant moderate correlation in male endurance athletes were found between VS and LVPWTd (*r* = 0.37, *p* = 0.01).

VS and LVM, IVSTd, LVPWTd or LVEDD, rVS, and LVEDDI showed no significant correlations in both male and female non-athletes. Thus, no significant correlation between VS and the systolic blood pressure was found in athletes and non-athletes.

## 4. Discussion

Continuous and long lasting workloads exert an overload on cardiac muscles and result in an exogenous hypertrophic pattern with normal ventricular walls and increased ventricle (especially LV) volume [25,26]. The impact of training on cardiac structure and function depends on the type, intensity, and duration of the activity, as well as previous physical activity engagement, genetics, and gender type [27,28,29]. Aortic dissection and rupture are occasional causes of sudden death in athletes, and incidences of such events proliferate with increasing aortic diameter in non-athletes too [5,6,9]. Classical normograms help assess aortic root diameter in the general population [23,30], yet there are no normograms for evaluating professional athletes. Therefore, the purpose of this study was to investigate aortic root adaptation to the workload and to determine if aortic root size and LV remodelling depend on physical load.

Results from this study indicated similar aortic annulus and sinus sizes to Boraita et al. [31]. In the present data, we observed q sinus size of 28.9 ± 2.36 mm and 32.38 ± 2.96 mm for female and male athletes, respectively, while sinus values were 27.19 ± 2.87 mm and 31.39 ± 2.72 mm for female and male non-athletes. In our study, significant differences were found among the female athletes and female non-athletes. Specifically, aortic root diameter at sinuses in female athletes was higher as compared to female non-athletes. The relative diameter of the aortic annulus was higher in female strength athletes compared to the endurance athletes. Our data confirms results previously obtained within Italian athletes [32]. Furthermore, it was shown that strength sports athletes had wider aortic root diameter compared to endurance sports athletes. We also compared basketball players with strength sports athletes. We expected that a large body surface area of the basketball players may have an effect to the relative values of the aortic root diameter. We found that the non-relative aortic diameter values of the basketball players tended to increase in comparison to the strength athletes, but the relative size of the aortic root diameter with the body surface area (and the aortic sinus and aortic annulus) was statistically significantly higher only for the female strength athletes compared to the female basketball players. Importantly, our study demonstrated the need to evaluate the aorta diameter changes according to relative aorta values.

In summary, our study revealed a significant moderate direct correlation between the aortic root diameter at sinuses of Valsalva and the size of the LV in Caucasian athletes. There was also a strong correlation between the former variables in female strength athletes. Based on the present data, we can conclude that as the athletes’ left ventricle mass and cavity increases, the diameter of the aortic root at sinuses increases. This said, these correlations were not found in non-athletes (without cardiovascular system impairment). It is important to note, however, that the aortic diameter enlargement at sinuses of Valsalva without LV remodelling is not associated with long-term physical load. Therefore, for female athletes, higher values of the aortic diameter at sinuses of Valsalva without the LV remodelling were found. As such, one could recommend female athletes to take part in sports other than the strength ones, or at least to reduce the amount of the maximal strength training.

There were several limitations to this study. First, we had a small size. Especially, low numbers of subjects were recruited in strength and endurance sport groups. Despite the small sample size through the groups were homogeneous according to physical exercise type. Secondly, there were potential confounds such as unequal time spent training and different workload intensity among athletes. Athletes had different trainers who used diverse training methodologies, possibly affecting adaptation to physical load, and this was not taken into account within the present study. Finally, athletes have recorded the amount of training at the moment when in echocardiography, hence the possibly of fluctuating training times in an athlete’s career, and this was not considered in the present study either.

We further confirm that echocardiography has to be performed, as well as aortic root and aortic valve morphology, which should be investigated in all competitive athletes as stated by Grazioli et al. [33]. To conclude, however, in the course of this investigation, we formulated a proposal for a potential course of action when enlarged aorta is observed. When enlarged aorta is observed, we propose to link the increase in the diameter of the aorta with the increase in LV size for athletes. The fact is that sport remodels LV is well-known; however, the present study is pioneering in that its results suggest both redesigned aorta and remodelled LV in athletic populations. In cases when the LV is not remodelled, but the enlarged aorta is present, we cannot talk about aortic remodelling due to sports.

## 5. Conclusions

It has been determined that the diameter of the aorta at sinuses is significantly higher in female athletes compared to female non-athletes. Aortic annulus’ relative diameter is greater in female strength athletes as compared to female endurance athletes. Female strength athletes also showed higher relative values of the aortic root (both at annulus and at sinuses) compared to female basketball players.

The diameter of the aortic root at sinuses had a positive correlation with the size of the left ventricle in athletes. Specifically, as the athlete’s LV mass and cavity increases, the diameter of the aortic root at sinuses also increases.

## Figures and Tables

**Table 1:** Baseline characteristics of study participant’s athletes and non-athletes.

	Female Athletes	Female Non-Athletes	*p* Value of Comparison of Female Groups	Male Athletes	Male Non-Athletes	*p* Value of Comparison of Male Groups
Age, years	22.78 ± 5.52	23.07 ± 6.13	*p* = 0.57	21.97 ± 5.41	21.49 ± 1.91	*p* = 0.28
Height, cm	173.14 ± 8.99	168.14 ± 4.90	*p* = 0.02	184.41 ± 9.04	183.44 ± 8.69	*p* = 0.62
Weight, kg	67.42 ± 11.55	64.43 ± 12.29	*p* = 0.30	80.17 ± 12.98	79.00 ± 15.77	*p* = 0.51
Body surface area, m^2^	1.8 ± 0.19	1.73 ± 0.18	*p* = 0.22	2.02 ± 0.19	2.00 ± 0.22	*p* = 0.50
Systolic blood pressure, mmHg	116.98 ± 11.82	113.93 ± 14.70	*p* = 0.51	124.09 ± 12.79	127.94 ± 13.98	*p* = 0.24
Diastolic blood pressure, mmHg	69.12 ± 7.59	68.57 ± 12.16	*p* = 0.94	73.92 ± 8.78	73.89 ± 11.95	*p* = 0.59
Resting heart rate, bpm	69.34 ± 10.98	74.14 ± 13.02	*p* = 0.28	68.18 ± 9.75	69.56 ± 7.44	*p* = 0.57

**Table 2:** Baseline characteristics of female endurance, strength, and basketball players.

	Endurance Athletes	*p* Value of Comparison of Endurance and Strength Athlete’s Groups	Strength Athletes	*p* value of Comparison of Strength Athlete’s and Basketball Players Groups	Basketball Players
Age, years	23.08 ± 5.69	*p* = 0.68	21.72 ± 4.34	*p* = 0.42	23.66 ± 4.56
Height, cm	175.41 ± 8.13	*p* = 0.01	165.22 ± 7.55	*p* = 0.002	179.25 ± 7.96
Weight, kg	49.25 ± 11.62	*p* = 0.03	60.89 ± 9.08	*p* = 0.01	74.67 ± 10.75
Body surface area, m^2^	1.83 ± 0.19	*p* = 0.03	1.67 ± 0.15	*p* = 0.003	1.93 ± 0.18
Years of professional training, years	11.63 ± 6.38	*p* = 0.68	8.78 ± 6.08	*p* = 0.42	12.75 ± 5.58
Systolic blood pressure, mmHg	116.94 ± 9.89	*p* = 0.94	117.11 ± 17.86	*p* = 0.60	120.00 ± 11.28
Diastolic blood pressure, mmHg	69.38 ± 7.59	*p* = 0.72	68.22 ± 7.97	*p* = 0.55	70.83 ± 9.00
Resting heart rate, bpm	69.16 ± 10.43	*p* = 0.82	70.00 ± 13.42	*p* = 0.51	67.92 ± 11.67
Hours of training, hours/week	8.05 ± 3.74	*p* = 0.30	7.83 ± 3,34	*p* = 0.22	7.33 ± 2.78

**Table 3:** Baseline characteristics of male endurance, strength, and basketball players.

	Endurance Athletes	*p* Value of Comparison between Endurance and Strength Athlete’s Groups	Strength Athletes	*p* Value of Comparison between Strength Athlete’s and Basketball Players Groups	Basketball Players
Age, years	20.36 ± 4.18	*p* < 0.001	25.38 ± 6.17	*p* < 0.001	18.83 ± 2.46
Height, cm	186.28 ± 8.93	*p* = 0.01	180.44 ± 8.07	*p* < 0.001	192.21 ± 6.44
Weight, kg	78.74 ± 11.62	*p* = 0.40	83.2 ± 15.30	*p* = 0.68	83.05 ± 11.65
Body surface area, m^2^	2.01 ± 0.18	*p* = 0.91	2.04 ± 0.22	*p* = 0.11	2.10 ± 0.18
Years of professional training, years	10.59 ± 5.62	*p* = 0.88	10.60 ± 6.11	*p* = 0.60	9.63 ± 3.76
Systolic blood pressure, mmHg	123.62 ± 12.81	*p* = 0.78	125.08 ± 12.95	*p* = 0.30	127.47 ± 13.63
Diastolic blood pressure, mmHg	72.64 ± 9.34	*p* = 0.09	76.64 ± 6.87	*p* = 0.05	70.53 ± 9.11
Resting heart rate, bpm	70.15 ± 10.62	*p* = 0.005	64.00 ± 5.83	*p* = 0.005	70.84 ± 9.03
Hours of training, hours/week	8.93 ± 4.03	*p* = 0.68	7.80 ± 2.99	*p* = 0.49	7.21 ± 2.18

**Table 4:** Subjects’ aortic root correlation with LV size.

	Female Athletes						Male Athletes				
			Strenght		Endurance		Strenght			Endurance	
CC, r	VS, mm	rVS, mm/m^2^	VS, mm	rVS, mm/m^2^	VS, mm	rVS, mm/m^2^	VS, mm	rVS, mm/m^2^	rVS, mm/m^2^	VS, mm	rVS, mm/m^2^
**LVM, g**	0.55 *p* < 0.001		0.76 *p* = 0.02		0.55 *p* < 0.001		0.49 *p* < 0.001			0.52 *p* < 0.001	
**LVEDDI, mm/m^2^**		0.49 *p* < 0.001		0.73 *p* = 0.03		0.48 *p* = 0.01		0.42 *p* < 0.001	0.43 *p* = 0.03		0.43 *p* < 0.001

Notes: LV—left ventricle; LVM—left ventricular mass; LVEDDI—LV end-diastolic diameter index; VS—diameter of aorta at the sinuses of Valsalva; rVS—VS relative index with body surface area; CC—correlation coefficient.
